# Assessment of Psychological Distress in Adults With Type 2 Diabetes Mellitus Through Technologies: Literature Review

**DOI:** 10.2196/17740

**Published:** 2021-01-07

**Authors:** Giulia Bassi, Silvia Gabrielli, Valeria Donisi, Sara Carbone, Stefano Forti, Silvia Salcuni

**Affiliations:** 1 Department of Developmental Psychology and Socialization University of Padova Padova Italy; 2 Fondazione Bruno Kessler Trento Italy

**Keywords:** type 2 diabetes mellitus, technology assessment, psychological distress, technology, review, mobile phone

## Abstract

**Background:**

The use of technological devices can support the self-management of individuals with type 2 diabetes mellitus (T2DM), particularly in addressing psychological distress. However, there is poor consistency in the literature regarding the use of psychological instruments for the web-based screening of patients’ psychological distress and subsequent monitoring of their psychological condition during digital interventions.

**Objective:**

This study aims to review previous literature on the types of psychological instruments delivered in digital interventions for assessing depression, anxiety, and stress in patients with T2DM.

**Methods:**

The literature review was conducted using the PsycINFO, CINAHL and PubMed databases, in which the following terms were considered:* diabetes mellitus, measure, assessment, self-care, self-management, depression, anxiety, stress, technology, eHealth, mobile health, mobile phone, device,* and *smartphone.*

**Results:**

In most studies, psychological assessments were administered on paper. A few studies deployed self-reporting techniques employing automated telephonic assessment, a call system for screening and monitoring patients’ conditions and preferences, or through telephone interviews via interactive voice response calls, a self-management support program leveraging tailored messages and structured emails. Other studies used simple telephone interviews and included the use of apps for tablets and smartphones to assess the psychological well-being of patients. Finally, some studies deployed mood rating scales delivered through tailored text message–based support systems.

**Conclusions:**

The deployment of appropriate psychological tools in digital interventions allows researchers and clinicians to make the screening of anxiety, stress, and depression symptoms faster and easier in patients with T2DM. Data from this literature review suggest that mobile health solutions may be preferred tools to use in such digital interventions.

## Introduction

### Background

After the diagnosis of type 2 diabetes mellitus (T2DM), people have to follow new lifestyles by changing their physical and psychological habits. According to several studies, T2DM is associated with significant psychological impairments, particularly depression, anxiety, and stress [1-4]. The prevalence of depression (22.4%) and anxiety (32%) in individuals with T2DM is considerably higher than that in the general population (10%), with a negative impact on the disease itself [5]. The prevalence of depressive symptoms in older adults with T2DM can be as high as 79.4% [6]. Indeed, some studies have reported that high levels of anxiety, depression, and stress tend to cause impairment in health-related quality of life and poor disease outcomes [6-9]. Factors that affect the quality of life of patients with T2DM include medical comorbidities [10], older age [11], female gender [12], and living in rural areas [10]. For instance, authors have suggested that poor glycemic control is associated with the onset of depressive symptoms [7,8] as well as diabetes distress [9], and these symptoms can improve with better glycemic control. In particular, diabetes distress is different from psychological distress, as the latter refers to a general state of emotional disturbance consisting of symptoms of depression and anxiety [13], whereas diabetes distress or diabetes-specific distress is a specific term that describes an emotional state where individuals experience stress, guilt or denial, and the burden of self-management due to diabetes itself. If such symptoms remain untreated, mild diabetes distress can result in severe diabetes distress and/or depression [14,15]. Moreover, individuals with T2DM who have reported symptoms of anxiety should be motivated to self-monitor glycemic levels while they are symptomatic [6]. Indeed, symptoms of anxiety during euglycemia—a normal level of sugar in the blood—would be suggestive of an anxiety disorder [6]. People with T2DM also presented with more stress symptoms compared with individuals who do not have this chronic disease [16]. Several studies have highlighted that stress symptoms can interact with the endocrine system, thereby involving physical attitude and nutritional behaviors, by increasing the capacity to control blood glucose [17]. Individuals with T2DM require continuous monitoring by health care professionals regarding the organic effects of diabetes; conversely, anxiety, depression, and especially stress symptoms often remain unrecognized and therefore untreated [18]. Previous studies have shown that these psychological symptoms increase the risk of more negative outcomes related to diabetes, such as glycemic control and impaired cardiovascular functioning [19]. Indeed, authors have suggested integrating psychological and medical care to address psychological symptoms and unhealthy habits (ie, sedentary lifestyle, poor diet), which often accompany depression, anxiety, and stress, as they seem to implicate benefits regarding the disease itself [6], the patients’ quality of life, and their psychological well-being. Therefore, early detection and prompt treatment of anxiety, depression, and stress symptoms can lead to a better medical prognosis and a better quality of life for individuals with T2DM. For instance, patients with this disease who have received psychological interventions have shown an increase in satisfaction with treatment [20]. Therefore, as a first step, it is important to identify valid tools that can assess the levels of anxiety, depression, and stress related to T2DM management to define the appropriate treatment. Within this framework, technological devices for supporting self-care in patients with T2DM are increasing globally [21]. In particular, these instruments are mainly focused on monitoring blood glucose and physical activities through several technological devices. The use of technological apps helps support people with T2DM in the management of their psychological distress and stress. However, although studies to date have shown promising results in the use of smartphone apps for diabetes management, there are inconsistent findings regarding the type of technological devices through which the best psychological instruments, developed as paper-and-pencil tools, should be delivered to achieve a more in-depth screening of patients’ psychological distress and thereby better outline the psychological intervention [22].

### Objective

This paper intends to identify and outline the types of technological devices through which psychological instruments should be delivered for an accurate assessment of diabetes-related psychological symptoms, such as stress, depression, and anxiety, in which technology represents support aid for self-care and self-management of T2DM. Indeed, a precise assessment of psychological symptoms through technologies in the field of diabetes is crucial to identify and understand problematic areas to better manage the disease itself, reduce such symptoms, and facilitate the management of the disease itself. Although the number of psychological instruments designed specifically for diabetes has increased, reviews of psychological instruments integrated into technological devices for assessing psychological symptoms are generally scarce among patients with T2DM. More specifically, the aim of this review is two-fold: (1) to summarize the types of technological devices used to administer self-report questionnaires for the assessment of psychological symptoms among individuals with T2DM, with a specific focus on the efficacy and usability of these tools and (2) to summarize the principal instruments as well as their psychometric characteristics to assess psychological symptoms related to T2DM, with a focus on symptoms of anxiety, stress, and depression, through the use of technology.

## Methods

### Study Design

This literature review sheds light on the types of technological devices through which psychological instruments were used to assess psychological distress among adults with T2DM. This review was conducted through the academic databases PubMed (360 articles), PsycINFO (165 articles), and CINAHL (239 articles) in which the following terms and their derivatives were considered during the search: diabetes mellitus, measure, assessment, self-care, self-management, depression, anxiety, stress, technology, eHealth, mobile health, mobile phone, device, and smartphone. More specifically, the terms self-care and self-management have been used as search words to find articles in which technological devices are mentioned. Moreover, according to the Cambridge Dictionary, the term device refers to a machine, for instance, a phone or a computer, which can be used to connect to the internet [23]. In this review, these search terms were used to identify devices suitable for assessing psychological distress in T2DM. We began the review with an examination of the types of technologies through which psychological questionnaires were administered and continued by examining the principal instruments for assessing psychological symptoms related to T2DM. We then concluded the review by providing directions for future work and clinical implications.

### Inclusion Criteria

The studies included in the review were in line with the following inclusion criteria: (1) presence of technological support for mental health assessment delivered to patients with T2DM; (2) studies with at least 60% of participants with T2DM, in order to have most people with only T2DM, the target population of this study; (3) studies that focused on depression, anxiety, stress, or other psychological symptoms in patients with T2DM; and (4) samples comprising adults aged between 18 and 70 years who may present with psychological distress, with a focus on depression, anxiety, and/or stress symptoms related to T2DM.

### Exclusion Criteria

Studies that met any of the following criteria were excluded: (1) absence of technological support for mental health assessment; (2) studies involving patients with other chronic conditions or primary diseases (eg, cardiovascular disease, cardiomyopathy, chronic kidney disease) or psychiatric disorders, according to the Diagnostic and Statistical Manual of Mental Disorders, 5th Edition [24]; (3) presence of individuals with the risk of T2DM onset; (4) studies that focused only on monitoring glycemic control or physical activity in patients with T2DM; (5) presence of cognitive dysfunction in patients with T2DM; (6) samples based only on individuals with type 1 diabetes mellitus; (7) studies that took into account samples of children and adolescents and/or parents supporting their children with diabetes; and (8) studies that focused on pregnant women with diabetes.

## Results

### Included Studies

On the basis of the inclusion and exclusion criteria, 17 articles were eligible for the literature review.

### Types of Technologies Through Which Tools Were Administered

Most studies (6 articles) were conducted in paper-based format—at baseline and follow-up—through written questionnaires provided by the staff or employing interviews before the technological intervention to evaluate the psychological distress and health-related quality of life of patients with T2DM [25-30]. Furthermore, 4 studies administered self-reports through the automated telephonic assessment (ATA), a call system for screening and monitoring that is tailored on patients’ conditions and preferences [31-34] or in 1 study through telephone interviews via the interactive voice response (IVR) call self-management support program (ie, tailored messages, structured email) [35]. In addition, in 2 studies, tools were administered through simple telephone interviews [36,37]. In 1 study, questionnaires were delivered through tablets available from a site to assess the psychological well-being of patients [38], whereas another study used a mobile app [39]. Furthermore, 3 studies were conducted using the mood rating scale, which consists of asking the patient “how do you feel?” through a tailored text message–based diabetes support system (ie, tablets, mobile phone) [38,40,41]. Specifically, the mood rating scale is integrated into the technology to evaluate moods and their patterns over time. The research question concerning the efficacy and usability of the tools has not found a clear and complete answer. It seems that this research question was not fully addressed in previous research but just *tangentially* touched on or inferred as a parallel finding.

### Advantages and Disadvantages of Technological Devices

The use of information and communication technology (ICT) apps in health care settings is increasing globally. Indeed, a useful way of communicating preventive methods to the population is through ICT [42,43]. This is motivated by an interest in facilitating active participation for people to self-manage their health as well as by the need to develop apps and platforms, which can be more cost-effective, compared with traditional approaches, and also to manage chronic conditions, such as diabetes [44,45]. For instance, the ATA or automated telephone communication systems is an app used to deliver both preventive health care programs and services to manage chronic conditions. Several studies have analyzed the ATA system for the management of diabetes [46,47], heart failure [48,49], coronary heart disease [50], and asthma [51] as well as for health-promoting methods, including dietary behavior [52,53] and physical activity [54,55]. ATA can deliver voice messages and gather health-related information from patients using voice recognition programs or touch-tone telephone [56], in addition to, or instead of, the telephone interaction between health professionals and patients. In particular, ATA has 3 subcategories: (1) unidirectional ATA, which enables one-way, noninteractive voice communication, including, for instance, interventions such as automated reminder calls to take medication; (2) the IVR system, which is the most common form of two-way real-time communication, allowing automated tailored feedback based on the monitoring of an individual’s progress, thereby allowing one-to-one interventions [56,57]; and (3) ATA with additional functions, namely ATA Plus, such as access to an expert to request support and ask questions via telephone or face-to-face meetings, and also the delivery of automated, nonvoice communications such as SMS text messages or email [58]. ATA—conceived as a data collection tool—presents several advantages compared with the classical face-to-face assessment [59], such as simplicity, anonymity, and low costs [56,60]. Therefore, ATA can allow access to health care systems 7 days a week for 24 hours a day, together with immediate feedback to the patient [61,62]. Indeed, studies reported higher levels of user satisfaction experience, suggesting that it is accessible for both patients and health care professionals [62]. Unlike face-to-face interactions, which can evoke socially acceptable answers, leading to the underreporting of stigmatizing behaviors and overreporting of socially desirable behaviors, ATA can elicit better self-reporting of specific and sensitive problems (eg, alcohol and substance use) and reduce self-reporting bias [63] as well as health care delivery costs [64,65]. On the other hand, ATA may also have disadvantages such as the difficulty in catching, interpreting, or responding to patients’ nonverbal answers to the interview questions [63,66]. Moreover, people with physical disabilities can have difficulty in using ATA [67], and some people can prefer face-to-face interaction rather than ATA [68]. In this framework, the classical telephone interviews, unlike the ATA system, can allow researchers and clinicians to obtain additional indications from the emphasis, intonation, hesitations, and the words used. However, as with the ATA system, they may have difficulty understanding the nonverbal responses. Telephone interviews also include limited telephone coverage in specific areas and lower response rates [69]. On the other hand, health professionals who use telephone interviews as assessment tools also have the opportunity to request a follow-up. Other digital solutions mentioned in this review are mobile phones, tablets, and desktop computers, in which mobile phone apps can allow real-time tracking of mood status, for instance, in patients with diabetes [70], anywhere and at any time, and they are particularly suitable for delivering immediate feedback, which makes them preferable to tablets and desktop computers. Moreover, besides being able to send voice and text messages, mobile phones present more advanced features, such as web searching, high-quality cameras, a GPS, and sound recording. Altogether with strong processors and operating systems, large memories, and high-resolution screens, mobile phones have turned into handheld computers. In particular, the use of mobile phones is increasing in health care settings (defined as mobile health [mHealth]), allowing health professionals to provide easy and rapid access to updated medical information [71,72]. Indeed, a great number of mHealth apps have become useful tools for health professionals, including health record maintenance and access, clinical software apps for suggestions within disease diagnosis, patient management and monitoring, clinical decision making, and medical training [71,73,74]. Moreover, mHealth has been found to support better clinical decision making and improve patient health outcomes [74,75]. On the other hand, one major issue concerns the security of health information delivered via mobile phones. mHealth adopts wireless atmospheric media to transmit data in the form of radio signals, which seem to be vulnerable to hackers and therefore to modification or distortion [76]; moreover, mHealth is closely networked with other wireless devices [77]. However, most professionals think that mHealth could significantly improve health care delivery processes, thereby improving patients’ psychophysical health. Indeed, mHealth interventions can reduce costs, save time, facilitate access to medical information, and provide a simpler and quicker way for patients and clinicians to send medical communications. Therefore, the adoption of mHealth improves the lifestyle, nutrition, behaviors, and quality of life of people with various diseases, particularly with chronic conditions. Thus, mHealth is increasingly considered to be one of the best digital solutions for the support of individuals in the management of their disease and the improvement of their health conditions [77].

### Principal Instruments to Assess Psychological Symptoms in T2DM in the Technology Field

Most studies included in the review reported only the name of the instrument, thereby not providing information regarding the psychometric properties of the tools, such as reliability, validity, data monitoring, contextual collection, and/or whether the instrument was specified for the population with diabetes [28,31,33-36,38,40]. In addition, the reasons for using such tools were not described in the analyzed papers; thus, it was not possible to ensure that the scales were used to address diabetes. In contrast, studies that administered the Diabetes Distress Scale-17 (DDS-17) [3] to evaluate diabetes-specific distress in the management of the disease reported that this tool shows good reliability and internal validity of the measure across independent samples [28,33,36,38]. This is the only scale that has been validated specifically for diabetes distress, focusing on problems that those patients may experience, such as emotional burden, interpersonal distress, or regimen-related distress [3].

Another instrument that is widely used to assess depressive symptoms through technologies is the Patient Health Questionnaire-9 (PHQ-9); indeed, 9 studies administered the above questionnaire [25-28,31-34,38]. The Centers for Medicare and Medicaid Services recommend the use of the PHQ-9 for home health care patients. The PHQ-9 was tested in primary care, demonstrating clinical relevance in relation to the Diagnostic and Statistical Manual of Mental Disorder-IV-Text Revision [24,78]. The PHQ-9 further comprises 2 components: symptoms and functional impairment assessment, which is useful for diagnosis, and a severity score, which is useful for selecting and monitoring treatments [26,27,32,38]. In addition, 1 study that administered the PHQ-8, a standardized and validated scale, showed its good reliability in assessing depressive symptoms [37]. Therefore, the PHQ represents a good tool to assess depressive symptoms in chronic diseases, including T2DM, as shown by its wide use. With regard to other psychological symptoms examined in the included studies, the tools used to assess anxiety symptoms were few, although the literature highlighted how anxiety could influence the chronic disease itself [79] to a higher degree than depressive disorders. In this context, 1 study administered the Hospital Anxiety and Depression Scale-14 items [80] to assess anxiety and depression symptoms [40], and 2 studies [30,35] evaluated depression symptoms using the Center for Epidemiological Studies Depression Scale [81]. Furthermore, 2 other studies [31,33] analyzed depression symptoms using the Hopkins Symptom Checklist Depression-20 [82]. With regard to the evaluation of the emotional distress experienced by people with diabetes, 2 studies [27,35] administered the Problem Areas in Diabetes [83]. Furthermore, 2 studies administered the Brief Symptom Inventory [84] to assess a wide range of psychological symptoms [33,36]. Stress symptoms were evaluated using a psychological scale through the mood rating; this was found to be interesting, as this scale is a technological modality to assess the nature of mood, thereby giving special importance to the monitoring instead of the screening of these symptoms [38,40,41]. The most used self-reports through technologies evaluating diabetes quality of life can be grouped into 3 categories: one refers to pain that can interfere with normal work and the other 2 categories refer to the physical and emotional symptoms related to the quality of life as assessed through the Medical Outcome Study Short-Form (MOS-SF) Health Survey 12 and 36 items [85,86], respectively, which are considered to be reliable and valid scales [26]. Overall, 6 studies administered the short version of the MOS [30,31,34,35,39,40] and 2 studies administered the longer version [25,38]. One study [26] evaluated the self-perception of quality of life through the EuroQol-5 Dimension [87], which has been tested and validated to capture the difference in the quality of life in patients with chronic diseases [28]. One study [29] analyzed the healthy self-management of the disease itself through the Health Education Impact Questionnaire [88], and it has also been validated in a primary health care context with patients with several chronic conditions, including diabetes [29]. Therefore, it seems that these studies used tools specifically targeted to patients with chronic diseases, including diabetes, to evaluate their quality of life as they had to change their physical and psychological habits. It is worth noting that one of the variables that emerged after the revision of the included studies was self-efficacy. Self-efficacy is a key variable in the proper management of diabetes within the health care setting [89]. Indeed, it represents the awareness and the perception that each individual has of their capacity to produce the desired results necessary to influence events, thereby affecting their lives [30]. Indeed, in the context of T2DM diabetes, a study found that higher glycemic control is associated with better self-efficacy and self-care behaviors [90].

A summary of the types of technological solutions through which patient screening and psychological assessment were conducted is presented in Multimedia Appendix 1 [25-41]. In addition, Textbox 1 shows the advantages and disadvantages of administering questionnaires in paper-and-pencil form versus those in digital form. Furthermore, all the questionnaires used to assess psychological symptoms in patients with T2DM are described in-depth in Multimedia Appendix 2 [3,79,82-90].

**Advantages and disadvantages of administering paper-and-pencil questionnaires versus administering questionnaires through digital solutions.**

Paper-and-pencil questionnaires

- Advantages
  More cost-effective when surveying data in small samples
  Could increase a working alliance because of human interaction
  Benefits people who do not have internet access
  More favorable format when it comes to longer questionnaires
  No digital skills are required to answer
- Disadvantages
  Lack of immediate data analysis
  Data entry is needed to store them in databases
  Handwritten responses could be difficult to interpret, especially when it comes to open-ended questions
  Needs longer data processing
  Printing and archiving of the questionnaires are needed
  There is the option of skipping questions
  Human errors can occur when updating the database

Questionnaires through digital solutions

- Advantages
  Save time for clinicians and researchers
  Faster in delivering the questionnaires
  Directly collect data on the web
  Furnish an immediate feedback
  Collect data from people around the world
  More ecological (ie, no printing and other costs at the point of completing setup)
  Better graphic layout (ie, not only color images and text, but also dynamic and interactive animation)
  Collect all the responses, thereby avoiding unanswered questions
  Allows longer answers to open-ended questions
  Gives more time to fill in the questionnaires
- Disadvantages
  Familiarity with digital devices is required
  Possible difficulties for data analysis derived from people filling-in the questionnaire multiple times, which would bias the results
  Lack of technological devices
  Technical problems
  Unreliable network

## Discussion

### Principal Findings

This study aimed to review past literature regarding the types of technological devices used to administer psychological instruments for assessing psychological distress (ie, depressive, anxiety symptoms) and stress in patients with T2DM. Assessing diabetes-related psychological symptoms can be challenging, due in part to the complexity of diabetes care. Indeed, diabetes self-care should be multidimensional, including treatment for both organic and psychological symptoms, whereas at the same time, it should use technology-based tools, which show good psychometric properties and are validated by samples of patients with chronic diseases.

First, studies seem to focus on the evaluation of psychological distress and diabetes-specific distress, particularly anxiety, depression, and stress symptoms. Indeed, instruments that measure symptoms of anxiety, stress, and depression related to T2DM, administered through technologies, were analyzed, and their efficacy and usability were evaluated. In this context, the psychometric properties of the instruments are prerequisites for an accurate assessment of psychological distress in patients with diabetes. Inadequate reliability and validity of the tools make it difficult to detect the psychological well-being of patients with diabetes and the impact of interventions on their well-being or quality of life. Here, the timing of the test can influence its reliability and validity, and therefore it needs to be taken into consideration. Within a longitudinal evaluation of an intervention, if the duration of the test was too short, participants could recall information from the first time they completed it, which could bias the findings. Alternatively, if its duration was too long, participants may have changed significantly, which could also bias the results [91].

In most studies, screening was conducted using written questionnaires at baseline and after a follow-up as well as telephone interviews (ie, simple telephone interviews, ATA calls, and IVR) to assess psychological symptoms [25-32,36,37].

Few studies have used digital solutions, such as mobile apps, tablets, and computers, to deliver psychological self-reports for intervention groups, even though they were investigating the psychological symptoms related to the disease itself [26,31,33,35-39,84]. In this context, the recent progress in technologies supports the ecological momentary assessment of mood, using mood ratings through mobile devices, outside the clinical environment. The mood rating scale can help to bypass issues associated with infrequent reporting of depressive symptoms and allows for a better representation of the dynamic nature of mood, which is often left unreported, and to better guide treatment planning [92,93]. For instance, delivering psychological instruments through technologies (eg, mobile phone apps) allows researchers to collect data directly from the web; thus, the wide use of the mood rating strategy would suggest saving time [94,95]. For instance, some studies have analyzed the feasibility of daily or weekly SMS text messages based on mood ratings, showing that mood ratings represent a valid monitoring strategy for patients with depression [70,96,97]. Mobile phone apps allow real-time tracking of mood status in patients with T2DM [70], anywhere and at any time, and they are particularly suitable for delivering immediate feedback (which makes them preferable to tablets and desktop computers). Moreover, they facilitate data collection in a more contextualized, pervasive, longitudinal, and reliable way, rather than using written questionnaires (preintervention and postintervention). In addition, they allow adapting the intervention to patients’ needs, supporting them in the management of their chronic disease. On the basis of other chronic diseases, mobile phone apps can provide psychoeducation [98], smoking cessation support [99], cognitive behavioral therapy [100], and support to caregivers [101]. Therefore, the identification of the appropriate psychological tools that can be embedded in digital devices, such as smartphones, could allow researchers and clinicians to conduct a screening of the level of anxiety, stress, and depressive symptoms in patients with T2DM in a faster and easier way.

Second, the PHQ-9 and the DDS-17 emerged as useful tools for the assessment of depressive symptoms in chronic diseases and for problems often experienced by these patients, such as emotional burden, interpersonal distress, or regimen-related distress [3]. Another widely used instrument is the MOS-SF-12, which assesses the physical and emotional symptoms related to the quality of life.

Moreover, considering the importance of the role of self-efficacy in the management of emotion-related diabetes, it is recommended that researchers and clinicians use specific tools to address self-efficacy in those patients. Of particular note is the fact that most studies used psychological tools in the standard paper format to assess the effectiveness of interventions without integrating them into digital solutions. Indeed, in this review, few studies delivered web-based questionnaires [38,39]. As an example, the Meru Health Ascend, a smartphone-based, therapist-supported intervention for depression and anxiety in patients with no chronic disease, delivered 2 validated scales for psychological distress (ie, PHQ-9 and the Generalized Anxiety Disorder-7) on a smartphone [102].

In this study, the integration of assessment instruments in digital solutions improved the assessment of depressive and anxiety symptoms. Thus, research suggests that mHealth interventions are functional ways of supporting the treatment of depression and anxiety symptoms [102].

### Limitations and Strengths

This review presents some limitations, as it included only papers in English, which limits the generalization of the findings. In addition, a limitation could be identified in the different implications regarding the use of technological devices from the age of 18 to 70 years. Older adults may be less familiar with the use of these devices. Future work should evaluate the effect of the use of technological devices among individuals of different ages. In the context of digital solutions, the disadvantage of smartphone-, tablet-, or computer-based apps is that they can be removed by the user; however, such device-based apps and other conversational agents can represent a valuable solution in administering psychological tools for the screening of patients, especially in emergency situations such as the SARS-CoV-2 pandemic. Furthermore, most studies do not provide information regarding the psychometric properties of tools, such as reliability, validity, and contextual collection. It could be important to include such characteristics to better understand the instruments used for assessing psychological distress and diabetes-specific distress. Another limitation is the noninclusion of videoconference calling as a digital solution, which especially during the COVID-19 lockdown represented the method of choice among practitioners in psychology and psychiatry for a remote assessment of mental health. Future studies should include these types of technological solutions to expand this literature review.

Finally, the focus of the review was only on psychological measures related to T2DM. In future works, one would recommend the integration of the assessment and monitoring of both organic and psychological symptoms in patients with T2DM.

On the other hand, this review also has some strengths. In addition to depression, the review considered all possible psychological symptoms related to T2DM.

### Future Development and Implications of the Study

This review highlighted implications that may have an impact on future research and clinical practice. In particular, the use of appropriate technological solutions to assess the psychological condition of patients can allow early detection of depression, stress, and anxiety symptoms, especially in chronic conditions as well as mental disorders [103]. In less severe cases, it might also help the deployment of mHealth interventions to support and improve depression, anxiety, and stress symptoms in T2DM to lower the burden for the health care system, in a stepped care approach [104]. Previous research found that mHealth solutions were well accepted by young adults [105], and further research is required to assess their acceptability by older adults with T2DM. Furthermore, because of the huge growth of mHealth apps, it would be useful to screen the levels of anxiety, stress, and depression symptoms in patients with diabetes, using appropriate psychological instruments. This screening could be able to identify whether the patient showed mild, moderate, or severe symptoms, thereby allowing clinicians to better set up interventions.

### Conclusions

In view of the large increase in the number of patients with diabetes globally, it is important to intensify efforts in the deployment of digital solutions for the accurate assessment of patients’ psychological condition. These instruments can be useful for clinicians and researchers to better monitor patients’ conditions. For example, the mood rating scale is widely used in studies to assess diabetes-related stress and depression. Therefore, the identification of appropriate psychological tools that can be deployed through mHealth solutions allows researchers and clinicians to screen for anxiety, stress, and depression in patients with T2DM in a faster and easier way. Moreover, data from the literature suggest that mHealth interventions are preferable to other types of digital interventions [101]. For instance, mHealth apps have recently been deployed in the field of psychology to deliver evidence-based treatments for depression and anxiety and to overcome barriers of face-to-face psychotherapy [101]. Therefore, this study can add to the scientific body of literature on the revision of valuable digital solutions in assessing and monitoring the mental health of patients, in which the traditional paper-and-pencil instruments can be delivered through digital solutions. Indeed, administering questionnaires through technological devices is a more cost-effective, time-efficient method for data gathering and for real-time tracking of mood status. Effective programs for chronic disease management should combine relevant information systems, with constant follow-up and targeted self-management for patients. In this way, ICT is incorporated in such a way as to provide accessible and convenient psychoeducational information as well as self‐management tools for people with long-term conditions. Finally, it is worth noting that ICT represents the only feasible way to assist and maintain health care, in chronic and nonchronic disease, in such a dramatic period as the actual pandemic, and thus gaining better knowledge and flexibility in the use of these methods becomes essential.

## Appendix

Multimedia Appendix 1 Articles from the literature review.

Multimedia Appendix 2 Description of the psychological instruments.

## Abbreviations

ATA: automated telephonic assessment
DDS-17: Diabetes Distress Scale-17
ICT: information and communication technology
IVR: interactive voice response
mHealth: mobile health
MOS-SF: Medical Outcome Study Short-Form Health Survey
PHQ-9: Patient Health Questionnaire-9
T2DM: type 2 diabetes mellitus
