# Supplementary material for: Assessment of Psychological Distress in Adults With Type 2 Diabetes Mellitus Through Technologies: Literature Review
Source: J Med Internet Res. 2021 Jan 7;23(1):e17740. doi: 10.2196/17740 (PMC7819779; doi:10.2196/17740)
Supplement: Multimedia Appendix 1 [file jmir_v23i1e17740_app1.docx]

**Table 1.** Articles from the literature review.

| Study | Sample characteristics | Psychological symptoms complying with the aim | Other psychological symptoms assessed in the papers | Instruments used | Types of technology used |
| --- | --- | --- | --- | --- | --- |
| Quinn et al [25] | - Older adults with T2DM^a^ (N=7) - M_age_=70.3 years - 57.1% females | Depression | Self-efficacy and health-related quality of life | - At baseline and at the end of the study—depressive symptoms: PHQ-9^b^ - At baseline and at the end of the study—self-efficacy: MOS-SF-36^c^ | Assessment through written questionnaires provided by staff |
| Quinn et al [26] | - Patients with T2DM divided into 2 groups (N=216; 108 patients received treatment as usual and 108 patients received a mobile phone–based SMS intervention) - Age: not applicable | Depression | Health-related quality of life | - At baseline and after 6 months—depressive symptoms: PHQ-9 - At baseline and after 6 months—health-related quality of life: EQ-5D^d^ | Assessment through written questionnaires |
| Quinn et al [27] | - Hispanic adults with T2DM divided into 2 groups (N=46; 25 intervention condition and 21 attention control condition) - M_age_=55.95 years - 67% females | Emotional distress and depression | — | At baseline and 12-month follow-up—distress (depressive) symptoms: PAID^e^ and PHQ-9 | Assessment through written questionnaires used for the control group and through computer-based version for the intervention group |
| Quinn et al [28] | - Adults with T2DM divided into 4 groups (N=163; 56 control-usual care, 23 coach only, 22 coach-PCP^f^ portal, and 62 coach-PCP portal with decision support) - M_age_=52.93 years - 81% males | Depression and distress | — | At baseline and follow-up interviews—distress (depressive) symptoms: PHQ-9 and DDS-17^g^ | Assessment through written questionnaires |
| Torbjørnsen et al [29] | - Adults with T2DM (N=110; of which 75 participants were in the intervention group) - M_age_=59 years - 56% females | Emotional distress | Health-related quality of life | Self-management of diabetes: Health Education Impact Questionnaire | Assessment through written questionnaires |
| Weinstock et al [30] | - Older adults with T2DM divided into 2 groups (N=1650; usual care and telemedicine) - M_age_=70.85 years - 60% females | Depression | Health-related quality of life | - Annual assessments—depressive symptoms: the CARE^h^ depression instrument - Annual assessments—health-related quality of life: MOS-SF-12 | Assessment through written questionnaires provided by personnel who were blinded to intervention status and were not involved in supporting the intervention |
| Ramirez et al [31] | - Adults with T2DM divided into 2 samples (N=117; sample for the first analysis and sample for second analysis) - M_age_=51.63 years - 84.44% females | Depression | — | - At baseline and after the intervention—depressive symptoms: PHQ-9 and HSCL-D-20^i^ - At baseline and after the intervention—health-related quality of life: MOS-SF-12 | Assessment through ATA^j^, a call system for depression screening and monitoring tailored to patient conditions and preferences |
| Ramirez et al [32] | - Adults with T2DM (N=444) - M_age_=52.59 years - 62% females | Depression | — | - At baseline and at 6, 12, and 18 months—depressive symptoms: PHQ-9 | Assessment through ATA, a call system for depression screening and monitoring tailored to patient conditions and preferences |
| Wu et al [33,34] | - Adults with T2DM Hispanic and Latinos divided in 3 groups (N=1406; 484 usual care, 480 supported care, 442 technology-facilitated care) - M_age_=53 years - 63% emales | - First article: depression and anxiety - Second article: depression | - First article: health-related quality of life (mental quality of life) - Second article: health-related quality of life | - First article:   - At baseline—distress (depressive and anxiety) symptoms: DDS-17 to screen for distress, PHQ-2 and PHQ-9, HSCL-D-20, and BSI-53^k^   - At baseline—health-related quality of life: MOS-SF-12, mental component summaries (PCS^l^ and MCS^m^) - Second article:   - At baseline—depressive symptoms: PHQ-9   - At baseline—health-related quality of life: MOS-SF-12 | In both articles there is the description of ATA, a call system for depression screening and monitoring tailored to patient conditions and preferences. *Amy* represent the digital voice of the automated call system who has the capacity to speak a natural voice |
| Aikens et al [35] | - Adults with T2DM (N=301) - M_age_=66.7 years - 97.0% males | Depression and distress | Health-related quality of life | - At baseline and after the intervention—distress (depressive) symptoms: Center for Epidemiological Studies Depression Scale-10 and PAID - At baseline and after the intervention—health-related quality of life: MOS-SF-12 | Assessment at baseline by telephone through the interactive voice response call self-management support program (tailored messages and structured email) |
| Rotheram-Borus et al [36] | - Women with T2DM (N=22) - M_age_=53 years (1 had type 1 diabetes mellitus ) | Anxiety and emotional distress | — | - Women were interviewed at recruitment, at 3 months N = 22 women reassessed), and at 6 months (N = 22 women)—anxiety symptoms: BSI | Assessment through interview provided in the Xhosa language by a research assistant for about 1 hour each to complete the evaluation measures |
| Trief et al [37] | - Adults with T2DM (N=268) - M_age_=56.8 years - 61.6% males | Diabetes distress and depression | Self-efficacy | - At baseline and at 4, 8, and 12 months: distress (depressive) symptoms: DDS-17 and PHQ-8 - At baseline and at 4, 8, and 12 months: self-efficacy: diabetes self-efficacy: 8-item scale | Telephone interviews |
| Holland-Carter et al [38] | - Adults with T2DM divided into 2 groups (N=904; 284 in standard care and 279 in weight watcher) - M_age_=55.1 years - 71% females | Distress and depression | Health-related quality of life | - At baseline and after the intervention—distress (depressive) symptoms: DDS-17 and PHQ-9 - At baseline and after the intervention—health-related quality of life: MOS-SF-36 - At baseline and after the intervention—general mood: mood rating, asking “How do you feel?” | - Questionnaires through tablets provided at the sites - Assessment through written questionnaires also at the follow-up visits |
| Munster-Segev et al [39] | - Adults with T2DM (N=7) - M_age_=55 years - 71% males | Emotional stress | Health-related quality of life | - No tools were used for emotional stress - The questionnaire was administered at baseline and at week 8. Measures were repeated at week 16—health-related quality of life: MOS-SF-12 | Assessment through mobile app *Serenita*, an interactive relaxation app that collects information through the finger touch of the patient and individualizes breathing instructions to modulate the stress level |
| Wayne et al [40] | - Adults with T2DM (N=97) - M_age_=53.2 years - 72% females | Depression and anxiety | Health-related quality of life | - At baseline and after 6 months—anxiety and depressive symptoms: Hospital Anxiety and Depression Scale-14 - At baseline and after 6 months—health-related quality of life and general mood: MOSSF-12 and mood rating, asking “How do you feel?” | Mood rating through mobile phone |
| Dobson et al [41] | - Adults with T2DM (N=42) - M_age_=45.7 years - 48% males | Distress | — | Mood rating | Mood rating through a tailored text message–based diabetes support |

^a^T2DM: type 2 diabetes mellitus.

^b^PHQ-9, PHQ-2, and PHQ-8: Patient Health Questionnaire.

^c^MOS-12 and MOS-36: Medical Outcome Study Short-Form Health Survey.

^d^EQ-5D: EuroQol- 5 Dimension

^e^PAID: Problem Areas in Diabetes, 20 items.

^f^PCP: primary care provider.

^g^DDS-17: Diabetes Distress Scale-17.

^h^CARE: the Comprehensive Assessment and Referral Evaluation

^i^HSCL-D-20: Hopkins Symptoms Check List Depression-20.

^j^ATA: automated telephonic assessment.

^k^BSI: Brief Symptoms Inventory.

^l^PCS: Physical Component summaries

^m^MCS: Mental Component summaries

—: Not Applicable
