# Supplementary material for: Assessment of Psychological Distress in Adults With Type 2 Diabetes Mellitus Through Technologies: Literature Review
Source: J Med Internet Res. 2021 Jan 7;23(1):e17740. doi: 10.2196/17740 (PMC7819779; doi:10.2196/17740)
Supplement: Multimedia Appendix 2 [file jmir_v23i1e17740_app2.docx]

**Multimedia Appendix 2.** Description of the psychological instruments

| **The Diabetes Distress Scale – 17 items (DDS-17) [3]** |
| --- |
| The DDS-17 is a self-report questionnaire used in clinical care and research, which assesses the diabetes-related emotional distress over the previous month. The questionnaire consists of 17 items scored on a 6-point Liker scale from 1 “no distress” to 6 “serious distress”. The DDS-17 comprises four subscales (i.e., emotional burden, physician-related distress, regimen-related distress, and interpersonal distress) and a total score. Higher scores indicate higher distress over the previous month. The DDS-17 has been translated in several languages available through the website of the questionnaire itself (i.e., https://behavioraldiabetes.org/scales-and-measures/#1448434304099-9078f27c-4106). The Cronbach’s alpha indicated a greater internal consistency with .93 for the total scale and ranging from .88 to .90 for the four subscales. |
| **The Patient Health Questionnaire-9 (PHQ-9) [79]** |
| The PHQ-9 is a short, well-validated questionnaire, which measures depressive symptoms based on Diagnostic and Statistical Manual of Mental Disorders, IV Edition-Text Revision (DSM-IV-TR) criteria for major depressive disorder, explained also in the DSM-5 Edition. The questionnaire is used for diagnosis, screening, monitoring and for determining the severity of the depression. Indeed, it has been administered in several studies regarding primary care settings. It is comprised of 9 items based on a 4-point Likert scale, from 0 “never” to 3 “almost every day”. Scores ranging from 0 to 27 in which scores from 0-4 indicate no depression, 5-9 minimal to mild depression, 10-14 moderate depression, 15-19 moderately severe depression and scores higher than 20 suggest severe depression. Therefore, a PHQ-9 score higher than 10 has been found to have 77-80% sensitivity and 92-94% specificity for a diagnosis of major depressive disorder (MDD) based on DSM-IV-TR. The PHQ-9 is available in English and over 30 other languages. The Cronbach’s alpha ranged from .86 to .89, showing a good internal consistency. |

| **The Hospital Anxiety and Depression Scale - 14 items (HADS) [82]** |
| --- |
| The HADS is a self-report questionnaire, validated in populations with cardiovascular disease. It allows to screen patients for psychological comorbidities in an efficient way. The questionnaire measures anxiety and depression symptomatology. The HADS comprises 14-items rated on a 4-point Likert scale, from 0 "absence" to 3 "extreme presence". The HADS include two scales: anxiety and depression with higher scores indicating higher levels of anxiety or depression. The HADS has been extensively validated in a variety of adult populations, including clinical and community samples, with well-documented excellent discriminant validity, construct validity, test-retest reliability, and internal consistency. |

| **Center for Epidemiological Studies Depression Scale (CES-D) [83]** |
| --- |
| The CES-D is a brief self-report questionnaire, which measures depressive symptoms severity in the general population. It consists of 20 items based on a 4-point Likert scale, from 0 "not at all" to 3 "a lot". More specifically, items 4, 8, 12 and 16 are developed to reflect positive affect and behaviour, and thus are scored in opposite order as follows: from 0 "a lot" to 3 "not at all". The CES-D scores' range from 0 to 60, in which higher scores suggest a greater presence of depressive symptoms. The CES-D shows good psychometric properties. |

| **The Hopkins Symptom Checklist-Depression- 20 (HSCL-20) [84]** |
| --- |
| The HSCL-D-20 incorporates the 13 items from the Hopkins Symptom Checklist Depression Scale and 7 additional items intended to assess criterion symptoms and improve responsiveness more completely. Each item is based on a 5-point Likert scale from 0 “not at all” to 4 “extremely”. higher scores indicate more severe symptoms. The HSCL-D-20 showed high internal consistency and test-retest reliability. |

| **The Problem Areas in Diabetes (PAID) [85]** |
| --- |
| The PAID is a self-report questionnaire, developed to measure emotional distress in people with Type 1 and Type 2 Diabetes Mellitus. It is composed of 20 items scored from 0 "not a problem" to 4 "serious problem". It showed to be responsive, thereby able to detect change when used in intervention studies. The PAID scores range from 0 to 100, where higher scores reflect a greater emotional distress. A score of 40 or above is indicative of severe emotional distress. |

| **The Brief Symptom Inventory (BSI) [86]** |
| --- |
| The BSI is a self-report questionnaire, which assesses several symptoms referring to nine dimensions equal to the Symptoms Checklist-90 dimensions, which are Somatization, Obsession-Compulsion, Interpersonal Sensitivity, Depression, Anxiety, Hostility, Phobic anxiety, Paranoid ideation and Psychoticism; and three global indices of distress: Global Severity Index, Positive Symptom Distress Index, and Positive Symptom Total. The BSI is composed by 53 items, based on a 5-point Likert scale, from 0 "not at all" to 5 "extremely". Test-retest reliability for the nine symptom dimensions ranges from .68 (Somatization) to .91 (Phobic Anxiety), thereby highlighting good psychometric properties. |

| **The Medical Outcomes Study Health Survey (MOS-36) [88]** |
| --- |
| The MOS-36 is a self-report questionnaire, and it was developed for the Medical Outcomes Study (MOS), a multi-year study of patients with chronic disease. The MOS-36 assesses health status and health-related quality of life in people's everyday life. The instrument includes 36 items based on a 5-point Likert scale, going from 1 "none" to 5 "extremely". It includes eight domains, which are Limitations in physical activities because of health problems, Limitations in social activities because of physical or emotional problems, Limitations in usual role activities because of physical health problems, Bodily pain, General mental health (psychological distress and well-being), Limitations in usual role activities because of emotional problems, Vitality (energy and fatigue), General health perceptions. It shows good psychometric properties. |
| **The Medical Outcomes Short Form Study Health Survey (MOS-SF-12) [87]** |
| The MOS-SF-12 is the brief version of the MOS-36, which investigated the same dimension of the MOS-36 |

| **EuroQol-5D (EQ-5D) [89]** |
| --- |
| The EQ-5D is a self-report questionnaire, which assess the health-related quality of life in the general population. The questionnaire consists of two parts: the first one includes 5 items referring to health aspects: Mobility, Personal care, Usual activities, Pain or discomfort, Anxiety or Depression. Each item is based on 3-point Likert scale, from 1 "absence" to 3 "presence" of moderate or serious problems. The second part of the questionnaire consists of a graduated scale from 0 to 100 (Visual Analogue Scale; SEA) in which people indicate their perceived health status. The EQ-5D demonstrates good psychometric properties. |

| **The Health Education Impact Questionnaire (heiQ) [90]** |
| --- |
| The heiQ is relevant for the assessment of psychological distress as it also evaluated an emotional distress component. It was developed in Australia and later adapted to multiple settings. Each of the 40 items is rated on a four-point Likert scale from 1 "strongly disagree" to 4 "strongly agree" and organized into 8 domains, which are The Positive and Active Engagement in Life, Health Directed Behavior, Skill and Technique Acquisition, Constructive Attitudes and Approaches, Self-Monitoring and Insight, Health Services Navigation, Social Integration and Support, Emotional Wellbeing (reversed scale). For all domains, high scores indicate a high level of self-management abilities, to the exception of emotional distress, where high scores reflect high distress. The heiQ demonstrates good psychometric properties. |
